# Reply to: A discrepancy of 10^7^ in experimental and theoretical density detection limits of aerosol particles by surface nonlinear light scattering

**DOI:** 10.1038/s42004-023-00904-7

**Published:** 2023-06-08

**Authors:** Yuqin Qian, Jesse B. Brown, Zhi-Chao Huang-Fu, Tong Zhang, Hui Wang, ShanYi Wang, Jerry I. Dadap, Yi Rao

**Affiliations:** 1 Department of Chemistry and Biochemistry, Utah State University, Logan, UT 84322 USA; 2 Department of Physics and Astronomy, Barnard College, New York, NY 10027 USA; 3 Stewart Blusson Quantum Matter Institute, University of British Columbia, Vancouver, V6T 1Z4 BC Canada

**Keywords:** Astrochemistry, Physical chemistry, Spectrophotometry, Infrared spectroscopy, Atmospheric chemistry

**replying to** A. Marchioro et al. *Communications Chemistry* 10.1038/s42004-023-00903-8 (2023)

Our recent work reported in situ observations of the chemical composition of laboratory-generated aerosol particle surfaces^1^. In a Matters Arising (MA) Article we are here replying to, Marchioro et al. raised an issue regarding the system’s detection limit in terms of particle density for the given particle sizes in our measurements. Their means for supporting this claim contains postulates based on a system not identical to ours. Our work^1^ does not claim that the vibrational sum-frequency scattering (VSFS) signals are generated explicitly from 40 to 100 nm diameter particles; instead, we stated, “The density of particles was estimated to be ca. 3.8 × 10^6^ cm^−3^, with a diameter centered at near 40 nm, and size distribution spanning from 10 nm to 300 nm.” We feel that due to incompatible comparisons, the MA Article made incorrect assumptions and conclusions about the detection limit of our system in terms of particle density based on the Rayleigh–Gans–Debye (RGD) theory. We believe that the issues raised in the MA Article we here reply to are inapplicable to our work as demonstrated below.

(1) Density-dependent non-resonant second-harmonic scattering (SHS) measurements are limited to background Hyper–Rayleigh signals of D_2_O: the MA Article showed non-resonant SHS with a high-repetition rate laser, which is not relevant to our study. The key difference between their system and ours is the fact that they use a liquid–liquid system while we use a liquid–gas system. Such differences are expected to modify the (1) physical-chemical environment, and (2) the molecular orientation and distribution at the aerosol-ambient interface. For their system,$\,{I}_{{total}}^{{SHS}}(N)={I}_{{D}_{2}O}^{{SHS}}+{I}_{{particle}}^{{SHS}}(N)$, where *N* is the density of the particles and *I* is the intensity at the double frequency. Although the SHS signals are linearly dependent on the particle density, the detection limit of non-resonant SHS signals is limited by the background signal from D_2_O. Thus, the non-resonant SHS of the 100 kHz system in the MA Article does not provide strong evidence against the detection limit of our system.

(2) Resonant sum-frequency scattering (SFS) measurements with a 1 kHz laser are limited to low collection efficiency: first, the collection efficiency in the MA Article (1–10 kHz) is at least 10× lower than that in our recent work (100 kHz laser system)^1^. In addition, the fluence of the visible pulse plays a vital role in signal intensity: ${I}_{{{{{{\rm{SFS}}}}}}}\propto {I}_{{{{{{\rm{Vis}}}}}}}{I}_{{{{{{\rm{IR}}}}}}}$, where *I*_*i*_ are the fluences of the relevant beams^2^. As this metric was not reported in the MA Article, it is unclear if it was accounted for in the calculations. Further affirmation of the effect of incident fluence is the difference between the IR fluence in our recent work versus those reported in Table 1 of the MA Article, which results in a 16-fold amplification of the SFS signal. Second, the MA Article investigated deuterated hexadecane droplets, with a 109 ± 1 nm average hydrodynamic radius, stabilized with 8 mM sodium dodecyl sulfate in D_2_O, with a path length of 200 μm. Based on the infrared transmission spectrum acquired for D_2_O by Carpenter et al.^3^, the transmission of the IR pulse energy is <18% at 2900 cm^−1^. The high absorption of D_2_O and the materials attenuates the IR pulse, resulting in a significant decrease in the generated SFS signals. On the other hand, our system consists simply of water droplets in nitrogen, for which the transmission of all relevant wavelengths involved in the SF process in our system is >99%. This contrast easily brings signals up by one to two orders of magnitude. Together, the signal levels in our case could be at least 10^3^–10^4^ higher than those in the MA Article. The use of a gas environment not only significantly improves the optical transmission but also removes contributions arising from interactions of the particle surface with its environment. Such interactions could substantially modify the surface nonlinear susceptibility of the particle as well as generate higher-order nonlinear optical contributions arising from, e.g., double-layer or field-induced effects.

(3) Size-dependence of nonlinear scattering signals: our group has been working on developing a theoretical understanding of our experimental results of VSFS from aerosol particles since it was first observed that they did not align with previous theories^1, 4^. However, there are still a few details that need addressing, so for now, we will continue to use the available theoretical works in order to address the remarks in the MA Article. Aerosol particles used in the original communication span the size range of 10–300 nm, with a density of 3.8 × 10^6^ cm^−3^. Our recent size-dependent SFS measurements have shown that the majority of the signal comes from particles from 10 to 300 nm in diameter, as discussed below. For this considered size range, the size parameter *ka* varies from 0.08 to 2.38, and consequently, the nonlinear scattering theory for small particle, which predicts a signal dependence of (*ka*)^6^ would not be applicable. Based on Figure 1B in the MA Article (reproduced in Supplementary Note 1 and Supplementary Fig. S1), it shows that a particle density of 10^14^ cm^−3^ would be required to observe an SFS signal from particles with 40 nm diameter. Supplementary Note 1 also shows how our experimental data would fit into this plot: the solid blue diamonds denote the size range for our experiments, whereas the open blue diamonds represent the proposed necessary density from the MA Article. It is shown that this highly contributing region between the solid blue diamonds overlaps with the range previously observed (red squares), which means that the density required for our experiments would be on the order of 10^10^ cm^−3^. The four orders of magnitude difference between the observed 10^6^ and supposed 10^10^ cm^−3^ values, the discussed discrepancy in Supplementary Note 1, is made up for by the advantages of pulse fluence, repetition rate, and optical transmission as discussed herein. Together, the signal level from our system is readily attainable.

**Fig. 1:**
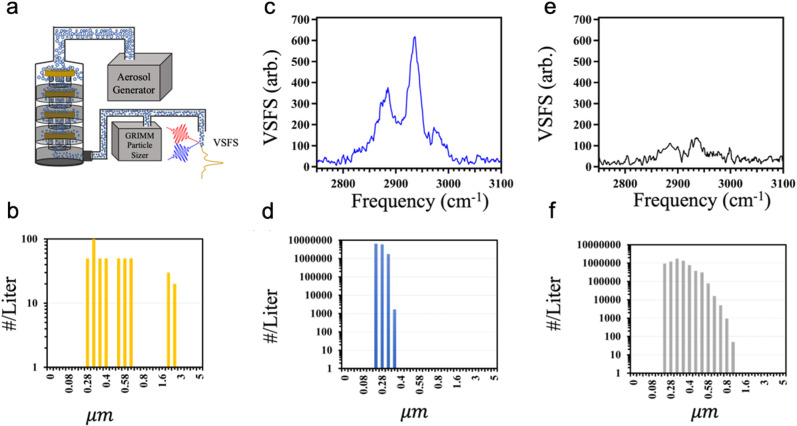
Size-dependence of vibrational sum-frequency scattering (VSFS) signals. **a** Schematic of the experimental setup. **b** Size distribution of particles from ambient air in our laboratory. **c** VSFS spectrum of particles with 10–350 nm diameter. **d** Size distribution of particles less than 350 nm after filtering. **e** VSFS spectrum of particles with diameters >265 nm. **f** Size distribution of particles >265 nm. All beams are horizontally polarized.

(4) The observed VSFS signal has negligible contributions from particles larger than 1 μm in our measurements^1^: additional experiments were conducted to demonstrate this fact using particles generated by the same methods as reported previously, using 6 M ethanol in 0.5 M NaCl^1, 4^. To check if our signals were from particles larger than 1 μm, we introduced an impactor (MOUDI™ 100-R, MSP Corp.) to filter out larger particles, as shown schematically in Fig. 1a and Supplementary Note 2, and Supplementary Fig. S2. The MOUDI™ is an industry standard for studying particles and their size distribution which has been well developed for decades^5^. It is noted that only particles larger than 200 nm in diameter were measured in this case (GRIMM Dust Monitor 1.100, Grimm Technologies Inc.), since we focused only on how large particles contribute to our observations. First, we tested the sensitivity of particle size measurements. Figure 1b shows particle distribution up to 5 μm from ambient air in our laboratory. Such measurements indicate that the particle sizer is capable of detecting tens of particles per liter in the given size range, as specified by the manufacturer. Second, we used the impactor to filter particles larger than 350 nm. The resulting VSFS spectrum and corresponding particle distribution are shown in Fig. 1c, d, respectively. Observing particles in this size range, we were still able to see a clear VSFS spectrum, with an intensity of 20% less than that without the filter. Third, we used the particle impactor to selectively decrease the density of smaller particles and introduce larger particles. Unlike the conjecture suggested in the MA Article, our VSFS signal was, in fact, lower for these size-selected particles, as shown in Fig. 1e. The corresponding size distribution data for this test are shown in Fig. 1f Moreover, we utilized a 90° VSFS collection geometry, which preferentially selects signals from particles much smaller than the relevant wavelengths^6^. Together, the VSFS signal observed in our recent work was not from particles larger than 1 μm and comes from the broad distribution of aerosol particle sizes of 10–300 nm.

(5) Crystal formations on the aerosol surface are unlikely^1^: the MA Article suggests that our VSFS signals might also be explained by a semicrystalline layer on the surface of the aerosol droplets. However, the humidity surrounding the aerosols was greater than the deliquescence point of NaCl aerosol particles^7–9^. The high solubility of the organics used in our work also invalidates the claim of a crystalline presence^1^. In addition, n-alcohols (C = 1–4) used in our follow-up report^4^ provided similar VSFS signal intensities to those in our recent work, and we believe that there is no reason to expect alcohol species to form crystalline or semicrystalline layers on the aerosol surface under high-humidity conditions. Previous experiments by Qian et al. investigating the effects of salt concentration and relative humidity on particle surface properties demonstrate some of the possible effects that crystalline formation can have on SHS intensity^7^. Figures 3B and D in the original article compare the SHS signals from aerosol particles at different humidities, above and below the deliquescence point of NaCl, respectively. Observing the first points for the different isotherms, we find that there is an approximate 2× increase in the SHS intensity for crystalline NaCl particles over the aqueous species. It is, however, unclear if the dye molecules used in these experiments were crystalline under these conditions. Based on this observation, and the humidity surrounding the particles in our recent work, it is unlikely that crystalline formation contributed to our SFS signals.

(6) Signal-to-noise ratio (SNR): the SNR is an effective measure of the sensitivity of an instrument. However, it is highly dependent on the laser system, detection system, molecular system, ambient environment, and, most importantly, spectral acquisition time; and is therefore typically an intrasystem quantity^10^. For this reason, a more suitable comparison would be to calculate the generated photons/s and then complete the comparison by accounting for the quantum efficiency of a given detector at the appropriate wavelength. It is necessary to find a common ground with which to compare the instrumental systems that accounts for all variables, especially acquisition time.

In summary, our measurements of the vibrational features from laboratory-generated aerosol particle surfaces have been carried out in a rigorous, meticulous, and well-calibrated fashion, and thus, we believe are accurate. Although our preliminary results are promising, there is still yet much to be explored about the fundamental physics behind the interesting observations from aerosol particles. At this point, we do not yet have a clear understanding of the physical and chemical mechanisms governing nonlinear scattering from water droplets in the air. To gain further insights into the scattering mechanism in our system, we plan to carry out angle-dependent VSFS experiments for a narrow distribution of aerosol particle sizes in the future. Meanwhile, a common ground needs to be established as a benchmark for other researchers who wish to conduct SFS experiments on aerosol particles, a common ground that is not optically and chemically different.

The VSFS signals in our case originate from the broad distribution of aerosol particle sizes of 10–300 nm, i.e., a system of nanoparticles suspended in the gas. We believe that the reasons for our SFS signal being above the calculated detection limit in the MA Article are valid and significant, and affect the detection limit as follows: 4× difference in IR fluence contributes to 16× difference in signal level (assuming that the fluence difference in the visible beam is similar, however, this was not reported); repetition rate of 100 not 1–10 kHz gives 10–10^2^ increase in signal; study of particles in a dispersive medium leads to strong IR absorption which gives 10–10^2^ lower signals; using RGD scattering theory based off *ka* gives signal proportionality to *a*^3^ not *a*^6^ which leaves the discussed discrepancy of 10^4^ cm^−3^ which is accounted for by the other factors above. Based on these estimates, the observation of a clear SFS signal from aerosol particles with the specifications in our recent work is quite feasible.

## Supplementary information

Supplementary Information

## Data Availability

All relevant data for this work have been uploaded and can be accessed through 10.6084/m9.figshare.22684888^11^.

## References

[1] Qian Y (2022). In situ analysis of the bulk and surface chemical compositions of organic aerosol particles. Commun. Chem..

[2] de Beer AGF, Roke S, Dadap JI (2011). Theory of optical second-harmonic and sum-frequency scattering from arbitrarily shaped particles. J. Opt. Soc. Am. B.

[3] Carpenter AP, Christoffersen EL, Mapile AN, Richmond GL (2021). Assessing the impact of solvent selection on vibrational sum-frequency scattering spectroscopy experiments. J. Phys. Chem. B.

[4] Qian Y, Brown JB, Zhang T, Huang-Fu Z-C, Rao Y (2022). In situ detection of chemical compositions at nanodroplet surfaces and in-nanodroplet phases. J. Phys. Chem. A.

[5] Marple VA, Rubow KL, Behm SM (1991). A Microorifice Uniform Deposit Impactor (MOUDI): description, calibration, and use. Aerosol Sci. Technol..

[6] Dadap JI, Shan J, Eisenthal KB, Heinz TF (1999). Second-Harmonic Rayleigh scattering from a sphere of centrosymmetric material. Phys. Rev. Lett..

[7] Qian Y, Deng G-h, Lapp J, Rao Y (2019). Interfaces of gas–aerosol particles: relative humidity and salt concentration effects. J. Phys. Chem. A.

[8] Tang IN, Tridico A, Fung K (1997). Thermodynamic and optical properties of sea salt aerosols. J. Geophys. Res. Atmos..

[9] Cruz CN, Pandis SN (2000). Deliquescence and hygroscopic growth of mixed inorganic−organic atmospheric aerosol. Environ. Sci. Technol..

[10] Skoog, D. A., Holler, F. J. & Crouch, S. R. *Principles of Instrumental Analysis* (Cengage Learning, 2017).

[11] Qian, Y. et al. *Comments Aerosols*. figshare.com (2023).

