# Supplementary material for: Reply to: A discrepancy of 107 in experimental and theoretical density detection limits of aerosol particles by surface nonlinear light scattering
Source: Commun Chem. 2023 Jun 8;6:115. doi: 10.1038/s42004-023-00904-7 (PMC10250303; doi:10.1038/s42004-023-00904-7)
Supplement: Supplementary file 1 — Supplementary Information [file 42004_2023_904_MOESM1_ESM.pdf]

Supplementary Information

Reply to: A discrepancy of 10^7 in experimental and theoretical density detection limits of aerosol particles by surface nonlinear light scattering

Yuqin Qian[1#], Jesse B. Brown[1#], Zhi-Chao Huang-Fu[1], Tong Zhang[1], Hui Wang[1], ShanYi Wang[1,2], Jerry I. Dadap[3], and Yi Rao[1]*
[1]Department of Chemistry and Biochemistry, Utah State University, Logan, UT 84322, United States
[2]Department of Physics and Astronomy, Barnard College, New York, NY 10027, United States
[3]Stewart Blusson Quantum Matter Institute, University of British Columbia, Vancouver, Canada V6T 1Z4
***Corresponding Author:** Yi Rao (yi.rao@usu.edu)
# Those authors contributed equally to this work.

Supplementary Note 1

Reproduction of Figure 1B from Ref 1

Figure 1B from Ref 1 was reproduced to ensure accurate interpretation of the presented data with actual numerical values.[1]

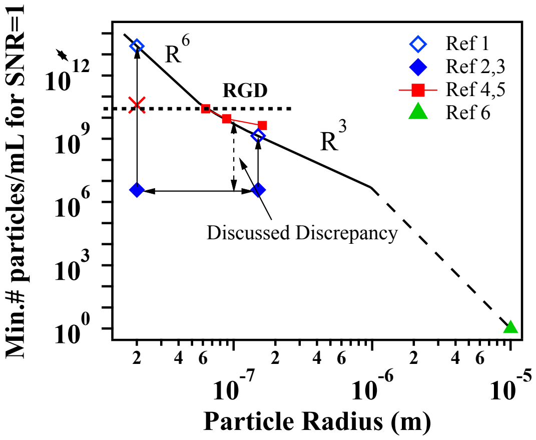

Figure S1. Reproduction of Figure 1B from Ref 1. The black solid and dashed lines represent the detection limit (SNR = 1) proposed in Ref 1.[1] The solid and open blue diamonds represent the experimental size range for Ref 2 and 3 and the proposed necessary density in Ref 1, respectively.[2, 3] The red squares represent experimental data from Ref 4 and 5.[4, 5] The green triangle represents the necessary size distribution of the large particles.[6] The discussed discrepancy gives the 10^4 difference between the observed and proposed signals, as discussed in the main text. The dotted horizontal line indicates that the red square experimental data were observed from densities on the order of 10^{10} cm^{-3}.

Supplementary Note 2

Aerosol Filtering by Particle Impaction

A micro-orifice uniform deposit impactor (MOUDI™ 100-R, MSR Corp.)[7] (Schematic in Figure 1, Photo in Figure S1) was used to selectively filter aerosols from the polydisperse stream of particles generated by the atomizer (TSI 3076). Such generated aerosols were produced as described previously.[2, 3] To selectively filter aerosols, the impactor uses stacked impaction plates allowing the user to successively eliminate smaller and smaller aerosol particles. As the concerns raised by Roke and coworkers explicitly consider contributions from larger particles,[1] we chose to use a particle sizer, which is capable of detecting just a few particles per liter for diameters greater than 250 nm (GRIMM Dust Monitor 1.100, Grimm Technologies Inc.). The particle sizer was used to measure the density and size distribution of the remaining particles after exiting the impactor. VSFS experiments were conducted as described previously,[2, 3] using the filtered stream of aerosols which exited the impactor. It is noted that passing the aerosol stream through the impactor without impaction plates resulted in a ~20% VSFS signal decrease, which was used as the benchmark for subsequent experiments.

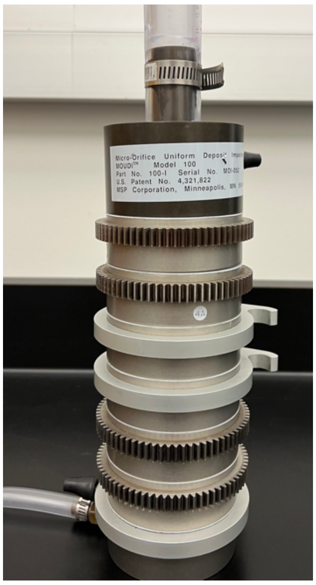

Figure S2. Photo of the micro-orifice uniform deposit impactor (MOUDI™) used in experiments. A MOUDI™ was used to filter sizes of aerosol particle for size-dependent experiments.

Supplementary References.

(1) Marchioro, A.; Golbek, T. W.; Chatterley, A. S.; Weidner, T.; Roke, S. Surface nonlinear light scattering of nanoscopic objects: Experimental and theoretical determination of aerosol particle density detection limits - a discrepancy of 107 *Communications Chemistry* **2023**, in press.

(2) Qian, Y.; Brown, J. B.; Huang-Fu, Z.-C.; Zhang, T.; Wang, H.; Wang, S.; Dadap, J. I.; Rao, Y. In situ analysis of the bulk and surface chemical compositions of organic aerosol particles. *Communications Chemistry* **2022**, *5* (1), 58. DOI: 10.1038/s42004-022-00674-8.

(3) Qian, Y.; Brown, J. B.; Zhang, T.; Huang-Fu, Z.-C.; Rao, Y. In Situ Detection of Chemical Compositions at Nanodroplet Surfaces and In-Nanodroplet Phases. *The Journal of Physical Chemistry A* **2022**, *126* (23), 3758-3764. DOI: 10.1021/acs.jpca.2c03346.

(4) Marchioro, A.; Bischoff, M.; Lütgebaucks, C.; Biriukov, D.; Předota, M.; Roke, S. Surface Characterization of Colloidal Silica Nanoparticles by Second Harmonic Scattering: Quantifying the Surface Potential and Interfacial Water Order. *The Journal of Physical Chemistry C* **2019**, *123* (33), 20393-20404. DOI: 10.1021/acs.jpcc.9b05482.

(5) Bischoff, M.; Biriukov, D.; Předota, M.; Roke, S.; Marchioro, A. Surface Potential and Interfacial Water Order at the Amorphous TiO2 Nanoparticle/Aqueous Interface. *The Journal of Physical Chemistry C* **2020**, *124* (20), 10961-10974. DOI: 10.1021/acs.jpcc.0c01158.

(6) Macias-Romero, C.; Nahalka, I.; Okur, H. I.; Roke, S. Optical imaging of surface chemistry and dynamics in confinement. *Science* **2017**, *357* (6353), 784-788. DOI: 10.1126/science.aal4346 (acccessed 2023/03/05).

(7) Marple, V. A.; Rubow, K. L.; Behm, S. M. A Microorifice Uniform Deposit Impactor (MOUDI): Description, Calibration, and Use. *Aerosol Science and Technology* **1991**, *14* (4), 434-446. DOI: 10.1080/02786829108959504.
